# Primary dysmenorrhea and quality of life among university nursing students in Vietnam

**DOI:** 10.3389/fgwh.2025.1639543

**Published:** 2025-12-05

**Authors:** Ba Nha Pham, Tuyet Minh Luu, Thi Ngan Phan, Minh An Ho, Tien Hoang Nguyen

**Affiliations:** 1 Women’s Health Center, Vinmec Times City International Hospital, Hanoi, Vietnam; 2 Department of Nursing-Midwifery, Hanoi Medical University, Hanoi, Vietnam; 3 Faculty of Pharmacy, Hanoi University of Pharmacy, Hanoi, Vietnam

**Keywords:** primary dysmenorrhea, quality of life, menstrual cycle disturbance, caffeine, PD

## Abstract

**Background:**

Primary dysmenorrhea (PD) is a common gynecological condition affecting many young women, especially university students. It can have significant effects on daily activities and quality of life. This study aims to describe the prevalence and some factors related to PD among female nursing students at Hanoi Medical University and evaluate its impact on students quality of life. The goal is to provide data for developing treatment and prevention strategies for PD in the community, particularly among female university students.

**Methods:**

A cross-sectional descriptive study was conducted on all female nursing students at Hanoi Medical University from January 15th to 31st, 2024. A simple random sampling method was used to select 341 participants. The data were collected using a paper survey and analyzed using SPSS software. The chi-square test was used to compare proportions, and difference rates along with 95% confidence intervals (CIs) were applied to evaluate associations between variables.

**Results:**

The majority of the students were aged 20 years or older, accounting for 72.8%. Most students (73.9%) reported menarche between the ages of 13 and 18. Out of 341 students, the prevalence of PD was 78.6%. Among them, 68.7% reported moderate to severe pain. PD was significantly associated with a family history of menstrual pain (*p* < 0.001), irregular menstrual cycles (*p* = 0.02), and frequent caffeine consumption (*p* = 0.03). Quality of life, assessed using the Q-LES-Q-SF questionnaire, was significantly lower in all domains among students with PD compared with those without menstrual pain (*p* < 0.05).

**Conclusion:**

PDis highly prevalent among female nursing students and negatively affects their quality of life. Several modifiable and non-modifiable factors are associated were found to be significantly associated with PD. The findings underscore the need for targeted interventions and further analytical studies to improve reproductive health and well-being among female students.

## Introduction

Menstrual disorders are a significant public health concern, with dysmenorrhea being one of the most prevalent gynecological conditions affecting women of reproductive age (1, 2). Dysmenorrhea is associated with considerable physical discomfort, emotional distress, and reduced quality of life, often resulting in absenteeism from school or work and decreased academic or occupational performance. Global prevalence rates of dysmenorrhea range from 45% to 95%, with severe symptoms reported in 2%–29% of cases (3, 4). In various regions, high prevalence has also been reported—74.5% in Asia (5), 71.1% in Australia (6), and 62.1% in Taiwan (7). In Southeast Asia specifically, studies have shown a prevalence of 91.3% among female medical students in Indonesia, 85.7% among adolescent girls in Malaysia (8, 9). Despite these high prevalence rates across Southeast Asia, research on this issue among Vietnamese female students remains limited. These data reflect the substantial burden of dysmenorrhea throughout the region and emphasize the need for increased attention to menstrual health, especially in young female populations.

Dysmenorrhea is classified into two types: PD which occurs in the absence of identifiable pelvic pathology, and secondary dysmenorrhea, which is associated with underlying gynecological disorders such as endometriosis or uterine fibroids (1, 2). PD typically begins during adolescence, within 6–24 months following menarche (10). It is characterized by cramping lower abdominal pain that begins just before or at the onset of menstruation and may persist for up to 72 h (2). The pain is usually midline and may radiate to the lower back or thighs (10). Associated symptoms frequently include nausea, vomiting, dizziness, fatigue, headaches, and sleep disturbances (3).

Quality of life refers to an individual's overall perception of their physical health, psychological state, level of independence, social relationships, and interaction with the environment (11). Among university students—particularly those in demanding health-related fields such as nursing—PD may significantly impair quality of life, impacting academic performance, daily functioning, emotional well-being, and social engagement (12).

Several studies have demonstrated the high prevalence of PD and its adverse effects among female university students. For example, a cross-sectional study conducted in 2018 among 376 female medical students at King Saud University in Saudi Arabia reported a prevalence of 80.1%, highlighting the condition's substantial impact on both quality of life and academic performance (13). Nevertheless, despite the growing international literature, evidence from Vietnam remains scarce. Nursing students, due to their intensive academic and clinical workload, may be particularly vulnerable to the effects of PD.

Therefore, the present study aims to determine the prevalence of PDand identify associated factors among female nursing students at Hanoi Medical University. The findings are expected to inform the development of appropriate interventions and policy strategies for menstrual health management in university settings. Ultimately, this study seeks to contribute to improving physical and mental health outcomes, academic success, and overall quality of life among female university students.

## Materials and methods

### Study design and setting

This cross-sectional study was conducted at Hanoi Medical University, Vietnam, from January 15 to January 31, 2024. A total of 341 female undergraduate students enrolled from the first to the fourth academic year, aged between 19 and 24 years, were included in the study.

### Eligibility criteria

#### Inclusion criteria

Female students aged 19–24 years who voluntarily agreed to participate and provided complete responses to the questionnaire.

#### Exclusion criteria

Students with a known history of chronic medical conditions or gynecological disorders such as endometriosis, fibroids, or pelvic inflammatory disease.

### Data collection tool

Data were collected using a structured, self-administered paper-based questionnaire comprising 34 items divided into three main sections:

• Section I: General demographic and academic characteristics of participants.
• Section II: Menstrual history, characteristics of primary dysmenorrhea, and associated factors.
• Section III: Impact of PDon various aspects of quality of life (QoL), including physical, emotional, and academic domains.

PD was identified based on participants’ responses to two screening questions: (1) “Do you experience menstrual pain during your menstrual cycles?” and (2) “Have you ever been diagnosed with any of the following conditions?”. A list of conditions was provided to help rule out secondary causes, including pelvic inflammatory disease, endometriosis, adenomyosis, fibroids, and other gynecological conditions.

The questionnaire, assessed using the Q-LES-Q-SF instrument, has been standardized into Vietnamese and approved by both the Scientific Committee and the Ethics Committee of Hanoi Medical University.

The survey was completed anonymously. No personal identifying information such as names, contact details, or addresses was collected to ensure confidentiality and privacy.

### Statistical analysis

All data were reviewed for completeness and consistency prior to analysis. Data entry and statistical analyses were performed using the Statistical Package for the Social Sciences (SPSS), version 25.0.

Descriptive statistics were used to summarize participant characteristics and outcome variables. Categorical variables were presented as frequencies and percentages, while continuous variables were described using mean, standard deviation (SD), minimum, and maximum values.

The chi-square (*χ*^2^) test was employed to examine associations between categorical variables. Odds ratios (ORs) and 95% confidence intervals (CIs) were calculated to estimate the strength of associations. An OR of 1 indicated no association, OR > 1 indicated a positive association, and OR < 1 indicated a negative association. A *p*-value of < 0.05 was considered statistically significant.

## Results

A total of 341 female nursing students, aged 19–24 years and spanning from first to fourth year, completed the survey questionnaire. Among them, 279 students (81.8%) reported experiencing primary dysmenorrhea, while 62 students (18.2%) reported no symptoms of dysmenorrhea (Table 1).

**Table 1:** Characteristic of students PD (*n* = 341).

Characteristic	*n*	%
Age		
Younger than 20 years	73	27.2
20 years or older	265	72.8
Experiencing PD		
Yes	279	81.8
No	62	18.2
History of secondary dysmenorrhea or gynecological disease		
Yes	11	3.2
No	330	96.8

The age of the first menstrual cycle among students was mainly in the group 13–18 years was 73.9%, and the group of ages 9–12 was 17,8%. The average age of first menstruation was 12.1 ± 1.3 years old. Most of the students have regular menstrual cycles ranging from 21 to 35 days, accounting for 54.5%. The remaining 122 students have irregular cycles, with the time between cycles being either less than 21 days or more than 35 days, accounting for 45.5%. The average duration of menstruation is 5.4 ± 1.0 days. The majority of students participating in the study have a moderate menstrual flow, with those having heavy and light menstrual flow (representing 78.7%, 12.7%, and 8.6%), respectively. Students participating in the study with a family history of dysmenorrhea (mother, grandmother, or sister) account for 35.8%. More than half of the students experienced dysmenorrhea in the first two years after their first menstrual cycle. The timing of the pain is as follows: on the first day of menstruation, which accounts for 69.8% (*n* = 187); one day before menstruation, 20.1% (*n* = 54); and more than two days before menstruation, 6% (*n* = 16). The pain typically lasts for one day (42.2%) or for two days or more (33.2%).

Most students participating in the study reported that they do not smoke (96.1%) or consume alcohol (97%), while nearly half (46.1%) stated that they do not exercise. When analyzing potential risk factors for primary dysmenorrhea, a significant association was found between caffeine consumption and the occurrence of dysmenorrhea. Specifically, 27% of students reported consuming at least 250 ml of caffeine daily for a minimum of three days per week, and among these, the prevalence of dysmenorrhea was significantly higher (89.9%) compared to non-consumers (*p* < 0.05). Other variables such as frequency of physical exercise days per week, smoking, alcohol consumption, daily sleep duration did not show statistically significantly associations with the presence of dysmenorrhea (Table 2). Additionally, student with irregular menstruation was also reported to have a higher rate of dysmenorrhea compared to regular menstruation (87.1% vs. 76.8%), with the difference being statistically significant (*p* < 0.05). The amount of menstrual flow per cycle also showed a significant association with dysmenorrhea (*p* < 0.05). Furthermore, students with a family history of dysmenorrhea experienced higher rates of the condition compared to those without such a history. Univariate logistic regression analysis revealed that irregular menstrual cycles (OR 2.04; 95% CI, 1.13–3.7), a family history of dysmenorrhea (OR 3.29; 95% CI, 1.55–6.95), and caffeine consumption (OR 2.51; 95% CI, 1.18–5.32) were significantly associated with PD among female nursing students.

**Table 2:** Some factors related to PD (*n* = 341).

Characteristic	Menstrual pain (*n*, %)		Total (*n*, %)	*P* value	OR (95% CI)
	Yes (268)	No (62)			
Living habits					
Number of days exercising per week					
None	123 (80.9)	29 (19.1)	152 (46.1)	*p* = 0.116	1.02 (0.445–2.343)
1–2 days	106 (81.5)	24 (18.5)	130 (39.4)		0.98 (0.420–2.294)
3 days or more	39 (81.3)	9 (18.7)	48 (14.5)		1
Smoking					
Yes	1 (50)	1 (50)	2 (0,6)	*p* = 0.526	4.37 (0.27–70.925)
No	258 (81.4)	59 (18.6)	317 (96.1)		1
Previously	9 (81.8)	2 (18.2)	11 (3.3)		0.97 (0.205–4.61)
Alcohol consumption					
No	260 (81.3)	60 (18.7)	320 (97.0)	*p* = 0.921	1
Yes	8 (80)	2 (20)	10 (3.0)		0.92 (0.19–4.46)
Daily sleep duration					
<4 h	5 (83.3)	1 (16.7)	6 (1.8)	*p* = 0.698	1.1 (0.21–10.152)
4–7 h	180 (79.6)	46 (20.4)	226 (68.5)		1.41 (0.747–2.677)
>7 h	83 (84.7)	15 (15.3)	98 (29.70)		1
Menstrual cycle characteristics					
Age at menstruation onset					
9–12 years old	89 (80.9)	21 (19.1)	110 (33.3)	*p* = 0.8	0.97 (0.541–1.741)
13–18 years old	179 (81.4)	41 (18.6)	220 (66.7)		1
Number of days of menstrual cycle per period					
<3 days	16 (84.2)	3 (15.8)	19 (5.8)	*p* = 0.927	1
3–7 days	246 (80.9)	58 (19.1)	304 (92.1)		1.125 (0.097–13.036)
>7 days	6 (85.7)	1 (14.3)	7 (2.1)		1.415 (0.167–11.979)
Logistic regression was performed to determine significant predictors of PD among participants.					
Family medical history of menstrual pain					
Yes	96 (91.4)	9 (8.6)	105 (100)	*p* = 0.004	
No	172 (76.4)	53 (23.6)	225 (100)		OR 3.29 (1.55–6.95)
Caffein consumption					
No	188 (77.6)	53 (22.4)	237 (73.0)	*p* = 0.038	OR 2.51 (1.18–5.32)
Yes	80 (89.9)	9 (10.1)	89 (27.0)		
Menstrual cycle regularity					
Regularity	146 (76.8)	44 (23.2)	190 (57.6)	*p* = 0.033	OR 2.04 (1.13–3.7)
Irregularity	122 (87.1)	18 (12.9)	140 (42.4)		

Most students with PD have lower average quality of life scores across all domains, with a total average score of 40.41 compared to 51.22 for the control group without dysmenorrhea. Additionally, physiological needs (sexual activity), economic conditions, and living conditions did not show statistical significance (*p* > 0.05). One-way ANOVA testing between the average total quality of life score and the level of dysmenorrhea showed a significant difference in the average quality of life scores across different pain levels, with statistical significance (*p* < 0.05). In students with PD (*N* = 268), higher pain levels (assessed using the VAS scale) are associated with lower average quality-of-life scores**.**

## Discussion

Results from a study involving 341 nursing students at Hanoi Medical University reveal that the prevalence of PD was found to be 78.6%, indicating a substantial burden of this condition in Vietnamese female medical students. This rate is comparable to findings from other Southeast Asian countries, such as Indonesia, where Situmorang et al. reported a PD prevalence of 71.8% among medical students, and Malaysia, where Azhary et al. found a prevalence of 83.5% among adolescent girls in North Borneo (8, 9). These figures highlight that PD is a pervasive issue across the region, particularly among young women in academic settings. This finding is closely aligned with PD rates reported in various cross-sectional studies conducted in different countries, including 84.2% in Lithuania (14), 85.1% in Palestine (11), 83.6% in Northern Ghana (12), and 84.1% in Italy (15). This indicates that PD is a common public health issue that needs appropriate attention. However, some studies report lower prevalence rates, such as 66.3% among nursing students in Southern of Spain (16), 60% among 2,721 women in Canada (17). Differences in prevalence rates between studies may be attributed to factors such as sample size and the age group of females selected.

The average age at menarche was 13.1 ± 1.3 years, with the earliest onset at 10 years and the latest at 17 years, which aligns with most studies both domestically and internationally. According to Trang Thi Nguyen (18), the average age of first menstruation was 13.81 years, and Huong Thu Thi Trinh (19) reported it as 13.3 years. Other global studies suggest an average first menstruation age about 12.2 years (14). 75.4% of the students experience menstrual pain lasting 1–2 days or more and the duration of menstrual pain can last 24–48 h depend on each cycle, but it is rare to have a cycle with no pain at all (1, 17), these findings are similar to those reported in some studies. Common symptoms related to PDidentified in this study are consistent with those in a study conducted among Mexican college students, where the main symptoms associated with dysmenorrhea were lower abdominal pain (93%), discomfort (50%), and depression (48%) (20).

Results from (Table 3) show that 18.7% of students experience severe dysmenorrhea (VAS scores from 7 to 10), 50.7% experience moderate pain (VAS scores from 4 to 6), and 30.6% experience mild pain (VAS scores from 1 to 3), this result differs from some domestic studies. According Linh Vu Thi Thuy reported that 58% of patients had severe primary dysmenorrhea, 42% had moderate pain, and no patients had mild dysmenorrhea (21). This discrepancy may be due to the fact that the study by Linh Vu Thi Thuy involved patients seeking treatment at the Traditional Medicine Department of Hanoi Medical University Hospital, with pain levels moderate enough to require pain relief and medical consultation, hence no mild cases were reported. Fernandez, Hu, et al. (22) have reported a higher prevalence of PDin female students with a family history of the condition compared to those without such a history. Results from Table 4 show that individuals with a family history of dysmenorrhea have a 1.3 times higher prevalence of the condition compared to those without such a history. According to the study results (Table 4), individuals with irregular menstrual cycles are more likely to experience dysmenorrhea (87.1%), which aligns with previous research. A systematic review of 63 studies involving 64,286 women found that irregular menstrual cycles significantly increase the risk of primary dysmenorrhea. Our study also found higher rates of dysmenorrhea among those who frequently consume caffeine (Table 4), with a significant association [*p* < 0.05, OR = 2.51 (1.18–5.32)]. Thus, individuals who frequently consume caffeine have a 2.51 times higher risk of experiencing menstrual pain. Caffeine may cause uterine ischemia and exacerbate pain due to its vasoconstrictive effects. Research has shown a strong relationship between caffeine consumption and the development of pain, including dysmenorrhea (23).

**Table 3:** Quality of life according to the Q-LES-Q-SF scale.

Variable	Average score menstrual pain		*P* (T-test)
	Yes (268)	No (62)	
1. Physical health	2.58	3.58	<0.001
2. Mood	2.38	2.95	<0.001
3. Work (studies, part-time jobs)	2.81	3.42	<0.001
4. Household activities	2.73	3.32	<0.001
5. Social relationships	3.06	3.48	<0.001
6. Ability to self-care in daily life	3.06	3.73	<0.001
7. Physiological needs	3.0	3.0	0.631
8. Economic conditions	3.42	3.45	0.678
9. Living conditions	3.34	3.48	0.061
10. Ability to move without feeling dizziness, imbalance, or falling	3.15	3.74	<0.001
11. Visual ability for work or recreation	2.79	3.39	<0.001
12. Overall perception of health	2.40	3.27	<0.001
Overall score	40.41	51.22	<0.001
Distribution of quality of life scores by level of dysmenorrhea	*N*	Average total score	*P*
Mild pain	81	43.18	<0.001
Moderate pain	137	39.84	
Severe pain	50	36.63	
Total	268	40.41	

**Table 4:** Demographic factors of students PD (*n* = 268).

Characteristic	*n*	%
Age at first menstruation		
9–12 years	89	26.1
13–18 years	179	73.9
Regularity of the menstrual cycle		
Regular (21–35 days)	146	54.5
Irregular (<21 days or >35 days)	122	45.5
Duration of menstruation (days)		
<3 days	16	6.0
3–7 days	245	91.4
>7 days	6	2.6
Blood volume		
Mild (1–2 sanitary pads completely wet)	23	8.6
Moderate (3–4 sanitary pads completely wet)	211	78.7
Severe (5–6 sanitary pads completely wet)	34	12.7
Family history of primary dysmenorrhea		
Yes	96	35.8
No	172	64.2
Onset of PDsymptoms		
At first menstrual cycle	92	34.3
6–24 months after menarche	80	29.9
2–3 years after menarche	52	19.4
4 years after menarche	28	10.4
Other	16	6.0
Timing of menstrual cramps onset		
On first day of menstruation	187	69.8
1 day before menstruation	54	20.1
≥ 2 days before menstruation	16	6.0
Other	11	4.1
Duration of pain		
Few hours	57	21.3
1 day	113	42.2
≥2 days	89	33.2
Other	9	3.4
Symptoms accompanying menstrual cramps		
Pain in lower belly	260	97.0
Lower back pain	182	67.8
Tired	162	60.3
Diarrhea	35	13.1
Headache	34	12.7
Nausea	32	12.0
Insomnia	30	11.2
Dizzy	28	10.5
Other	4	1.5
Severity of menstrual cramps		
Mild	82	30.6
Moderate	136	50.7
Severe	50	18.7
Pain relief methods		
Without medication	81	30.2
Taking medication	7	2.6
Both medication and other methods	46	17.2
No pain relief methods	134	50.0

Although a substantial proportion of female students reported experiencing PD, the findings revealed that only 2.6% of participants reported using pharmacological treatment for symptom relief. In contrast, 30.2% reported employing non-pharmacological approaches such as warm compresses, rest, or light physical activity. Despite the high prevalence of PD, the remarkably low rate of medical treatment-seeking suggests a possible normalization of menstrual pain, limited awareness of evidence-based management options, or restricted access to reproductive health services. These findings underscore the need for targeted educational interventions and improved access to menstrual health resources within university settings.

The findings support previous cross-sectional studies that show a clear decrease in quality of life associated with PD (23, 24). As pain severity increases, the overall quality of life score decreases, which is consistent with previous research involving a larger sample with varying pain levels, indicating a significant reduction in most quality of life domains with increasing pain severity (25). Symptoms related to dysmenorrhea among college or university students are severe, highlighting that PD is a serious public and gynecological health issue. Therefore, pain management is crucial for improving quality of life. Our findings suggest that measuring quality of life will be a useful indicator for assessing the effectiveness of different treatment methods for primary dysmenorrhea. Numerous studies indicate that PD is associated with various factors: early menarche, a family history of dysmenorrhea, heavy menstrual flow, smoking, and substance use are all risk factors for dysmenorrhea (26–28).

## Conclusion

This study found a high prevalence of primary dysmenorrhea (78.6%) among nursing students at Hanoi Medical University, with 68.7% reporting moderate to severe symptoms. Significant associated factors included a family history of dysmenorrhea, irregular menstrual cycles, and regular caffeine consumption.

Students with dysmenorrhea had lower quality of life scores across all domains, and higher pain levels were associated with poorer overall quality of life.

Educational and psychological support programs aimed at increasing menstrual health awareness and coping strategies may help reduce the impact of primary dysmenorrhea and improve students’ well-being.

## Data Availability

The raw data supporting the conclusions of this article will be made available by the authors, without undue reservation.
